# Biomass expansion factor and root-to-shoot ratio for Pinus in Brazil

**DOI:** 10.1186/1750-0680-6-6

**Published:** 2011-09-24

**Authors:** Carlos R Sanquetta, Ana PD Corte, Fernando da Silva

**Affiliations:** 1 Department of Forest Science, Federal University of Paraná, Curitiba, PR, Brazil. Av. Prof. Lothário Meissner, 900, Jardim Botânico, Curitiba, Paraná, 80.210-170, Brazil; 2 Forestry Research Foundation of Paraná, Curitiba, PR, Brazil. Av. Prof. Lothário Meissner, 900, Jardim Botânico, Curitiba, Paraná, 80.210-170, Brazil; 3 Course student at National Research Institute of Amazonia, Manaus, AM, Brazil. Av. Prof. Lothário Meissner, 900, Jardim Botânico, Curitiba, Paraná, 80.210-170, Brazil

**Keywords:** allometry, carbon, regression, CDM, modeling

## Abstract

The Biomass Expansion Factor (BEF) and the Root-to-Shoot Ratio (R) are variables used to quantify carbon stock in forests. They are often considered as constant or species/area specific values in most studies. This study aimed at showing tree size and age dependence upon BEF and R and proposed equations to improve forest biomass and carbon stock. Data from 70 sample *Pinus *spp. grown in southern Brazil trees in different diameter classes and ages were used to demonstrate the correlation between BEF and R, and forest inventory data, such as DBH, tree height and age. Total dry biomass, carbon stock and CO_2 _equivalent were simulated using the IPCC default values of BEF and R, corresponding average calculated from data used in this study, as well as the values estimated by regression equations. The mean values of BEF and R calculated in this study were 1.47 and 0.17, respectively. The relationship between BEF and R and the tree measurement variables were inversely related with negative exponential behavior. Simulations indicated that use of fixed values of BEF and R, either IPCC default or current average data, may lead to unreliable estimates of carbon stock inventories and CDM projects. It was concluded that accounting for the variations in BEF and R and using regression equations to relate them to DBH, tree height and age, is fundamental in obtaining reliable estimates of forest tree biomass, carbon sink and CO_2 _equivalent.

## Background

CDM (Clean Development Mechanism) enables developing countries to participate in global efforts to reduce greenhouse gases (GHG) concentrations in the atmosphere and to accomplish the Kyoto Protocol commitments. However, few A/R CDM approved methodologies were available until recently and hence a small number of PDDs (Project Design Documents) were submitted to the CDM Executive Board (EB) of the UNFCCC (United Nations Framework Convention on Climate Change). One of the main constraints to proposing an adequate A/R methodology and applying it in a PDD is the difficulty in identifying and monitoring the complex biological relationships in a forest plantation as well as obtaining reliable estimates of biomass and carbon stocks. In recent years, several methodologies have been approved by the EB that allow us to obtain stock change estimates for large forest areas, but they are complex and difficult do apply.

There are different approaches to calculating biomass and carbon stocks in forests. These are mostly based on forest inventory information as well as various factors, referred to as biomass factors, or biomass equations, which transform diameter, height or volume data into biomass estimates [1]. The calculations can be obtained by direct and indirect methods [2]. The direct method involves destructive biomass weighing, whereas in the indirect method regression modeling is used to estimate biomass and carbon stocks from more easily-measured tree and stand variables, such as DBH (diameter at breast height), tree height (H) and age. The use of either the direct or indirect methods may provide information to construct a figure of CO_2 _removal for a CDM project for the duration of a crediting period.

Estimates of biomass and carbon stocks from bole volume and wood density generally require the application of a biomass expansion factor (BEF) or biomass equations to obtain the aboveground and total biomass [3-5]. Because biomass factors are easier to use than biomass equations they have been preferred. BEF is calculated from the ratio of aboveground biomass and bole biomass (defined by a merchantable measure or a minimum DBH). When belowground biomass is considered in the CDM project, root-to-shoot ratio (R) also should be taken into account. Calculation of R involves simply dividing the root biomass by the corresponding aboveground biomass.

BEF and R application may also vary from project to project. Single default values are often used, as in Kauppi et al. (1992) [6], Kauppi et al. (1995) [7], Lowe et al. (2000) [8], UN-ECE/FAO (2000) [9], FAO (2001) [10] and IPCC (2006) [11]. However, it is known that these factors may vary depending on the species to be planted, growth phase, and site index [12]. Therefore, calculations of BEF and R under specific conditions shall be preferred [13-15].

This study deals with the analysis of correlations of BEF and R with some typical tree inventory data (DBH, H and age) and the development of mathematical modeling relating BEF and R to the more easily obtained tree measurement variables. In this paper a comparison of three different approaches to estimate biomass and carbon stocks is carried out, (*i.e. *IPCC default values of BEF and R [11], average BEF and R from field data and BEF and R estimated from regression equations fitted from specific field data).

## Results and Discussion

### Descriptive Statistics

Table 1 shown below summarizes the descriptive statistics of the variables analyzed in this study. The average value of BEF calculated from the data was 1.47, meaning that the crowns of the pine species studied here represent on average 32% of the aboveground biomass and 27% of the total biomass, however the variability in this factor was remarkable, ranging from 1.09 to 3.74. As stated before, the mean default value of IPCC for pines growing in the tropics and in similar conditions of this study is 1.30, ranging from 1.2 to 4.0.

**Table 1:** Descriptive statistics for DBH, H, Age, BEF, and R

Statistics	DBH (cm)	H (m)	Age (years)	BEF	R
Mean	20.10	15.15	11.44	1.47	0.17
Standard Deviation	8.78	7.65	5.95	0.47	0.11
Minimum	1.91	2.35	2	1.09	0.05
Maximum	40.27	30.30	24	3.74	0.63
Coefficient of Variation (%)	43.68	50.50	52.01	31.97	64.71
Number of cases	70	70	70	70	70

In work by Levy et al. (2010) [16], for three conifer species in Great Britain, the authors found BEF values ranging from 1.04 to 2.32. In a study of carbon stocks grown in Western Europe, Liski et al. (2002) [17] found a mean BEF of 1.39, while Schroeder et al. (1997) [18] estimated a mean BEF of 1.25 for adult deciduous tree stands in the US. The IPCC (2006) [11] gives various other BEF figures from literature. However, no specific studies on BEF were found for Brazilian pine plantations or under similar conditions.

Regarding R, the mean value found in this study was 0.17, varying from 0.05 to 0.63. This means that on average, belowground biomass corresponds to 15% of the total biomass. The mean default value for pine in the Tropics is R = 0.32, according to IPCC, though a range of 0.24 to 0.50 in this ratio has been reported [11]. In the study by Levy et al. (2010) [16], researchers found an average R of 0.36; whereas in the study in western Europe by Liski et al. (2002) [17], mean R was 0.16. The IPCC reported other references on the matter, but literature is not available for pine plantations in Brazil.

It is noteworthy to mention that both BEF and R figures given by the literature are influenced by the methodology used in each case. Some authors adopt the definition of bole as the main tree trunk up to a minimum diameter. A BEF figure from bole of 4 cm minimum diameter is obviously rather smaller than another calculated from 10 cm minimum diameter. It is more problematic for broadleaf species that have sympodial growth than for pines with monopodial crown architecture. Similarly, R is affected by the depth and fine root dimension approach utilized. Deep root excavations and fine root dimension imply in greater root-to-shoot ratios given same conditions.

As can be seen later on in this paper, BEF and R are correlated with DBH, height, and age, but the relationship among them is hardly constant. On the contrary, as the trees grow and advance in age the contribution of foliage and roots to the total biomass diminishes in both *Pinus *species studied here, though it will be discussed at greater length later in this paper.

### Correlation between BEF, R, DBH, H, and Age

The correlation analysis performed on the biomass expansion factor and the root-to-shoot ratio, tree diameter, height, and age indicated that BEF and R have significant correlations with the tree measurement variables, as shown in Table 2. All correlation coefficients were negative for BEF and R versus tree size and age, indicating a decrease in both tree size and age.

**Table 2:** Correlation matrix between BEF, R, DBH, H and Age variables

Variable	DBH (cm)	H (m)	Age (years)	BEF	R
DBH (cm)	1	-	-	-	-
H (m)	0.922	1	-	-	-
Age (years)	0.869	0.960	1	-	-
BEF	-0.731	-0.724	-0.671	1	-
R	-0.679	-0.728	-0.707	0.528	1

The correlation matrix indicates that DBH is the variable more closely associated with BEF, followed by height and age, respectively, and that all of them have significant correlations. In contrast to the findings of this study, Levy et al. (2010) [16], Brown & Schroeder (1999) [19] and Lehtonen et al. (2004) [15] found that tree height has the greatest explanatory power in estimating BEF. In this study, the explanatory power of DBH and height were nearly the same. Therefore, DBH shall be preferred as the explanatory variable because it is easy to measure, less time consuming and higher precision compared to height.

On the other hand, R was more closely correlated with height, though age and DBH were also significant. BEF and R were also correlated with each other in this study, but the correlation coefficient was moderately smaller. The correlation between BEF and R seems reasonable, since trees need more root biomass to support a proportionally large crown.

### Modelling BEF and R from DBH, H, and Age

**Figure 1:**
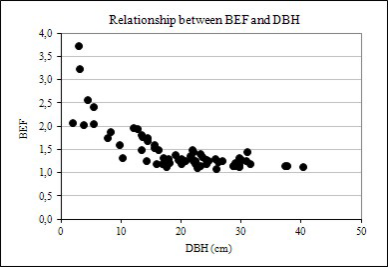
Relationship between BEF and DBH

**Figure 2:**
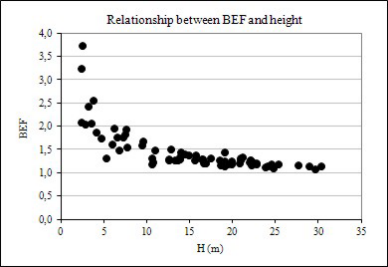
Relationship between BEF and height

**Figure 3:**
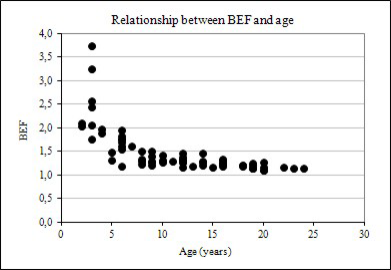
Relationship between BEF and age

The results showed that biomass expansion factor and root-to-shoot ratio vary considerably with tree size (DBH and height) and age. The relationships are shown graphically in Figures 1, 2 and 3. In evidence are the relationships between BEF and R with DBH, height, and age have a well-defined trend, following an negative exponential curve and that BEF and R decrease as DBH, height, and age increase. Thus, larger and older trees have proportionally less foliage and root biomass as compared to smaller and younger ones. A relative decreasing trend in crown (foliage + branches) and root biomass across a range of tree ages have been reported elsewhere, as in Kauppi et al. (1995) [7], Lehtonen et al. (2004) [15], Brown (2002) [20] and Fukuda et al. (2003) [21].

This trend may be explained by the physiological maturation of trees [22], which require greater photosynthetic biomass to promote faster growth in the earlier phases of this process and resource-use efficiencies of individual trees [23,24], as well as competition with neighbors and canopy closure [12,25,26], which causes shading of the tree crown and limitation of root expansion, and natural pruning [27]. However, a decrease in BEF and R with tree size and age tends to not take place after a given size and age, suggesting an asymptotic behavior due to stabilization of growth rate and tree maturation.

However, there was noticed heterogeneity of variance in BEF and R along tree size and age axes. This may be explained by the so-called Jensen's inequality, which describes how variance depresses the response variable in decelerating functions and elevates the response variable in accelerating functions [28,29]. The authors argued that Jensen's inequality provides a fundamental tool for understanding and predicting consequences of variance some direct effects of environmental variance in biological systems.

On the other hand, Magnani et al. (2000) [30] tried to explain the reduction of growth and biomass allocation with ageing from a physiological and hydraulic point of view. It has been shown that younger plants grow taller more quickly. Hence, they need proportionally more photosynthetic biomass than older ones. Some authors consider respiration is a key factor for limitation of foliage growth with ageing (Kira & Shidei, 1967; Barnes et al. 1998) [31,32]. However, Ryan et al. (2004, 2006) [33,34] rejected the traditional hypothesis that increased respiration of woody tissues forces a decline in above-ground net primary productivity by conducting an experimental test of causes of forest growth with stand age for *Eucalyptus*. According to the authors the decline was primarily caused by a decline in canopy carbon gain and secondarily by a shift in the annual partitioning of gross primary productivity to belowground allocation and foliage respiration.

**Figure 4:**
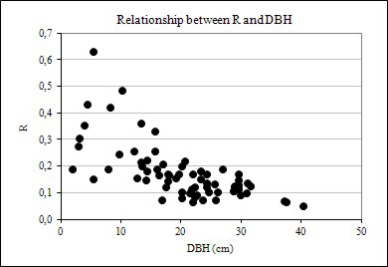
Relationship between R and DBH

**Figure 5:**
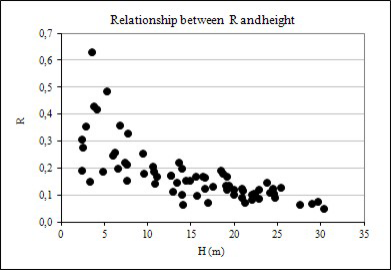
Relationship between R and height

**Figure 6:**
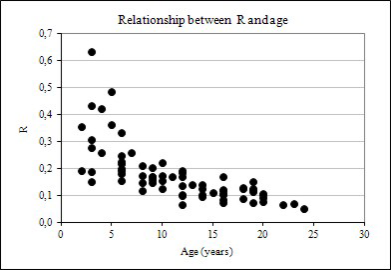
Relationship between R and age

Figure 4, 5 and 6 also suggests that R data dispersion is more pronounced in comparison to BEF, when these factors are plotted against DBH, height, and age, implying that forest inventory variables are more strongly correlated with BEF than with R, as seen later in this paper. Another feature of the graphs of BEF and R against DBH, H, and Age, is the greater dispersion for smaller and younger trees.

Similar results were also reported by Lehtonen et al. (2004) [15]. Soares & Tomé (2004) [35] found analogous trends and concluded from their study on effectiveness of biomass expansion factors that estimates of total stand biomass (aboveground and root biomass) should be derived from allometric equations and if an expansion factor must used then age-dependent BEFs are recommended. They also stated that the use of a constant BEF should be avoided because it yields inaccurate estimates.

Table 3 shows fitting statistics for the 12 models tested to estimate BEF and R from DBH, H, and Age. Equations for BEF, in general, resulted in better fit as compared to those for estimating R.

**Table 3:** Statistics of the fitted models to estimate BEF and R

Model	**β**_**0**_	**β**_**1**_	**β**_**2**_	**β**_**3**_	**β**_**4**_	**R^2^**_**adj**_	**S**_**yx**_	**S**_**yx**_**%**
MODELS FITTED TO ESTIMATE BEF								
1	3.9065	-0.3522	-	-	-	0.72	0.25	16.92%
2	3.6190	-0.3697	-	-	-	0.75	0.23	15.98%
3	3.2871	-0.3684	-	-	-	0.75	0.23	15.96%
4	2.2597	-0.0392	-	-	-	0.53	0.32	22.10%
5	2.1462	-0.0446	-	-	-	0.52	0.33	22.45%
6	2.0792	-0.0532	-	-	-	0.44	0.35	24.20%
7	2.5896	0.0301	-	-	-	0.62	0.29	20.02%
8	2.3734	0.0341	-	-	-	0.58	0.30	20.99%
9	2.3013	0.0421	-	-	-	0.50	0.33	22.94%
10	2.2313	-0.0215	-0.0347	0.0172	-	0.53	0.32	22.21%
11	3.0377	-0.2048	-	-	-	0.70	0.26	17.89%
12	3.5663	-0.9055	0.0296	-1.2032	0.3844	0.78	0.22	15.60%

**MODELS FITTED TO ESTIMATE R**								

1	0.5389	0.4037	-	-	-	0.35	0.09	48.84%
2	0.5887	0.5006	-	-	-	0.51	0.07	42.42%
3	0.5830	0.5576	-	-	-	0.51	0.07	42.15%
4	0.4836	-0.1082	-	-	-	0.43	0.08	45.53%
5	0.4662	0.1150	-	-	-	0.56	0.07	39.89%
6	0.4502	0.1215	-	-	-	0.55	0.07	40.47%
7	0.4017	0.0452	-	-	-	0.49	0.08	43.36%
8	0.3943	0.0609	-	-	-	0.57	0.07	39.72%
9	0.4003	0.0828	-	-	-	0.55	0.07	40.51%
10	0.3302	0.0008	0.0077	0.0020	-	0.53	0.07	42.14%
11	0.4800	-0.7298	-	-	-	0.53	0.07	41.45%
12	0.4105	0.0901	-0.1945	-	-	0.59	0.07	39.61%

**Figure 7:**
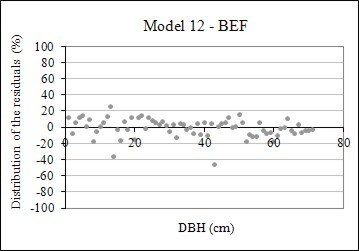
Graphical distribution of the residuals of the fitted models to estimate BEF using equation 12

**Figure 8:**
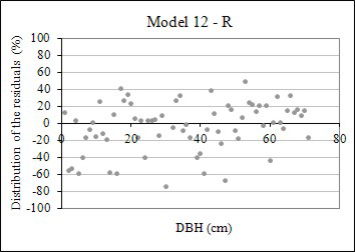
Graphical distribution of the residuals of the fitted models to estimate R using equation 12

The coefficient of determination for the 12 equations tested to estimate BEF ranged from 0.44 to 0.78, whereas for R stayed between 0.35 and 0.59. The standard error in the estimate for BEF equations ranged from 15.60 to 24.20%, whereas for R equations varied from 39.72 to 48.84%. When these two statistics of goodness of fit were taken into consideration, equation 12 was selected as the most accurate, though models 1, 2, 3, and 11 also presented similar performance. Equation 12 was also the most accurate for R estimation, though its mathematical formula differs from the BEF model, because both equations were fitted by means of stepwise regression. A graphical analysis of residuals performed on the 12 equations to estimate BEF and R confirmed those findings (Figures 7 and 8).

### Comparison of Different Approaches to Estimate Biomass and Carbon Stock

In order to demonstrate the impact of the various approaches to estimate biomass and carbon stock, an analysis based on the growth and yield simulations from SISPINUS software was performed. This computer program was developed by EMBRAPA - Brazilian Agricultural Research Center and is widely used by the forestry sector in the country and recognized as a reliable tool for simulating growth and yield of pine plantations throughout site conditions.

Growth and yield was simulated for a 1-hectare unthinned 18-year rotation stand, as described before. Based on the predicted volume (511.35 m^3^/ha), estimates of total biomass (dry aboveground + belowground biomass), carbon stock and CO_2 _equivalent were generated by the three different approaches, as described before (Table 4). The estimates using the IPCC [11] default BEF (1.30) and R (0.32) were 334.93 t/ha, 137.32 tC/ha, and 503.52 tCO_2_eq./ha, respectively. Using the mean values of BEF (1.47) and R (0.17) from the field data the estimates would be 335.69 t/ha, 137.63 tC/ha, and 504.66 tCO_2_eq./ha, respectively for total biomass, carbon stock, and CO_2 _equivalent. The difference between the estimates for these two approaches was less than 1%. However, when the calculations are made using regression equations the figures change drastically, to 251.32 t of total biomass, 103.04 t C/ha, and 377.82 t CO_2_eq/ha. Under this condition, the percentage difference in the estimates rises to 33%.

**Table 4:** Total dry biomass, carbon stock and CO_2 _equivalent in a one-hectare stand of *Pinus *spp. using constant IPCC default values for BEF and R, mean BEF and R and estimation by regression equations

Variable	**BEF and R default IPCC **	**Average BEF and R **	**BEF and R from regression equations **
**Total biomass (t/ha)**	334.93	335.69	251.32

**Carbon stock (t/ha)**	137.32	137.63	103.04

**CO**_**2 **_**equivalent (t/ha)**	503.52	504.66	377.82

-	+0.22		

**Difference (%) ** -		-33.27	

-	-33.57		

The results shown above indicated that the use of either default or mean BEF and R values may lead to a gross overestimation in biomass and carbon stocks, and consequently in CO_2 _removal by sinks in carbon sequestration projects for *Pinus *species in southern Brazil. Besides the overestimation of the environmental benefits of a project like this, it is also worth mentioning the financial implications of such overestimates. Even in small-scale CDM projects the consequences of such misestimates may be on the order of millions of US dollars. Therefore, project developers should be aware that using simplified methodologies may cause tremendous impacts throughout the offset planning table. Certainly the importance of modeling BEF and R in a reliable way is crucial for proposing a realistic carbon sequestration project.

## Conclusions

The following conclusions were obtained from the results and discussions of this research:

• For the *Pinus elliottii *and *Pinus taeda *plantations analyzed in this study Biomass Expansion Factor (BEF) and Root-to-shoot ratio (R) are strongly correlated with DBH and total height, and less strongly correlated with tree age. BEF and R decrease with DBH, height and tree age. The relationship between them suggests a negative exponential curve toward an asymptote with increasing BEF and R.

• In this study the highest R^2 ^and lowest Syx could be achieved using a combination of the following independent variables: DBH, height and age for BEF estimation; and DBH and height for R estimation;

• The use of default BEF and R should be avoided due to the great possibility of obtaining spurious results. For pine plantations in southern Brazil the overestimation may be over 33%;

• Finally, caution should be used by forest offset developers since the process of verification and certification may not confirm CO_2 _removals from sinks predicted by a project that uses either default or mean BEF and R values.

## Methods

### Field Data

The data used in this study came from 70 sampled *Pinus elliottii *and *P. taeda *individuals growing across southern Paraná State, Brazil. A direct method (weighing) was applied to obtain fresh biomass weight in the field. Belowground biomass was obtained after excavation and cleaning of the roots over 2 mm of diameter at 50 cm depth.

The sampled trees were representative of the local conditions and previously categorized by DBH and age classes. Girth at breast height of each sample tree was measured using ordinary metric tape, and values were converted to DBH for calculations and modeling. After felling, the top bole length of each tree was measured using tape and taken to be tree total height - H. Age was determined by tree ring counting and from historic records.

For the biomass weighing in the field, each tree was cut up into five biomass pools: bole, thick branches over 4 cm of diameter, thin branches under 4 cm of diameter, foliage, and roots. Biomass was determined following IPCC recommendations [11]. Each biomass pool was weighed using a mechanical balance with a 100 kg capacity and 100 g precision. A 500 g sample was taken from each biomass pool to determine dry weight, percentage of dry matter and carbon fraction in the laboratory. Carbon fraction was determined using a LECO-144 combustion chamber.

### Calculation of BEF and R

The biomass expansion factor (BEF) definition used in this study is based upon FAO (1997) [36], i.e., the ratio of aboveground oven-dry biomass of trees to oven-dry biomass of inventoried volume. Since for the conditions of this study the inventoried volume is the total bole, BEF becomes as shown by the following ratio:

$$BEF=\frac{W_{crown}+W_{bole}}{W_{bole}}=\frac{W_{aboveground}}{W_{bole}}$$

where:

*BEF *= biomass expansion factor (dimensionless);

*W*_*crown *_= tree crown dry weight (g), composed of foliage, thick and thin branches;

*W*_*bole *_= tree bole dry weight (g); and

*W*_*aboveround *_= *W*_*crown *_+ *W*_*bole *_(g).

The root-to-shoot ratio calculations were conducted using the formula below, as indicated by the [11], which defines R as the ratio of belowground (root) to aboveground biomass (shoot), as follows:

$$R=\frac{W_{root}}{W_{aboveground}}$$

where:

*R *= root-to-shoot ratio (dimensionless); and

*W*_*root *_= tree root dry weight (g).

### Statistical Analysis and Mathematical Modelling

Correlation coefficients of all variable combinations were calculated in order to understand the relationships among them and in turn to develop models to estimate BEF and R as functions of the more easily measured forest inventory variables (DBH, H, and Age). Twelve mathematical models were tested to estimate BEF and R from DBH, tree height and age as and presented in Table 5. Only linear models were chosen by the criteria of simplicity and easy-to-fit.

**Table 5:** Models tested to estimate BEF

MODELS FITTED TO BEF		MODELS FITTED TO R	
1	$BEF=\beta_{0}*DBH^{-\beta_{1}}$	1	$R=\beta_{0}*DBH^{-\beta_{1}}$
2	$BEF=\beta_{0}*H^{-\beta_{1}}$	2	$R=\beta_{0}*H^{-\beta_{1}}$
3	$BEF=\beta_{0}*AGE^{-\beta_{1}}$	3	$R=\beta_{0}*AGE^{-\beta_{1}}$
4	*BEF *= β_0 _+ β_1_*Ln(*DBH*)	4	*R *= β_0 _+ β_1_*Ln(*DBH*)
5	*BEF *= β_0 _+ β_1_*Ln(*H*)	5	*R *= β_0 _+ β_1_*Ln(*H*)
6	*BEF *= β_0 _+ β_1_*Ln(*AGE*)	6	*R *= β_0 _+ β_1_*Ln(*AGE*)
7	$BEF=\beta_{0}*EXP^{\left(-\beta_{1}*DBH\right)}$	7	$R=\beta_{0}*EXP^{\left(-\beta_{1}*DBH\right)}$
8	$BEF=\beta_{0}*EXP^{\left(-\beta_{1}*H\right)}$	8	$R=\beta_{0}*EXP^{\left(-\beta_{1}*H\right)}$
9	$BEF=\beta_{0}*EXP^{\left(-\beta_{1}*AGE\right)}$	9	$R=\beta_{0}*EXP^{\left(-\beta_{1}*AGE\right)}$
10	*BEF *= β_0 _+ β_1 _* *DBH *+ β_2 _* *H *+ β_3 _* *AGE*	10	*R *= β_0 _+ β_1 _* *DBH *+ β_2 _* *H *+ β_3 _* *AGE*
11	*BEF *= β_0 _+ β_1 _* *ln*(DBH * H * *AGE*)	11	*R *= β_0 _+ β_1 _* *ln*(DBH * H * *AGE*)
12	*BEF *= β_0 _+ β_1 _* ln *DBH *+ β_2 _* *DBH *+ β_3 _* ln*H *+ β_4 _* ln(DBH * H * *AGE*)	12	*R *= β_0 _+ β_1 _* *ln DBH *+ β_2 _* *ln H*

where: β_i _= coefficients.

The best-fit model was selected in accordance with the following criteria: smallest percentage standard error of estimate (Syx_%_), highest coefficient of determination adjusted to number of cases and coefficients (R^2^_adj_), and optimal performance in a graphical analysis of residuals.

### Comparison of Different Approaches to Estimate Biomass and Carbon Stocks

A comparison of three approaches to calculate BEF and R was performed. The first approach consisted of using constant IPCC values for pine plantations in the tropics, BEF = 1.30 and R = 0.32 [11], whereas the second took into account fixed values calculated from the average BEF and R of the trees sampled in this study. The third approach used size- and age-dependent estimates of BEF and R derived from the best-fit equation selected from the 12 models tested in this study.

The comparison was done by calculating CO_2 _equivalent from the three approaches, using volume estimation from a growth and yield simulator called SISPINUS [37]. Carbon dioxide equivalent was used for comparison because it is the variable used in carbon sequestration projects. The input variables for SISPINUS were: number of trees per hectare = 1,667; site index = 23 m (dominant tree height) at age 15 years; and rotation age = 18 years for an unthinned hypothetical stand. The output variable (total bole volume) was converted to carbon stock (t/ha) by multiplying wood density (0.3817 g.cm^-3 ^according to Sette JR et al. (2006) [38] to 0.41 carbon fraction of dry matter (tC.d.m.)^-1^, from lab determination, in accordance with IPCC nomenclature [11]. Carbon stock was in turn converted to CO_2 _equivalent (t/ha) by multiplying by the ratio 44/12 (1 mole C = 12, 1 mole O = 12, therefore 1 mole CO_2 _= 44).

## Competing interests

The authors declare that they have no competing interests. The views expressed in this publication are those of the authors.

## Authors' contributions

CRS, APDC and FS conceived, drafted the manuscript and developed the methodological approaches. All authors read and approved the final manuscript.

## References

[B1] Somogyi Z Cienciala E Makipaa R Muukkonen P Lehtonen A Weiss P Indirect methods of large-scale forest biomass estimation European Journal of Forest Research 2006 126 2 197 207

[B2] Sanquetta CR Corte APD Balbinot R Zilliotto MAB Carlos Roberto Sanquetta, Marco Aurélio Zilliotto Proposta metodológica para quantificação e monitoramento do carbono estocado em florestas plantadas Mercado de carbono: mercado e ciência 2006 1 Curitiba: UFPR 120 150

[B3] Johnson WC Sharpe DM The ratio of total to merchantable forest biomass and its application to the global carbon budget Can J For Res 1983 13 372 383 10.1139/x83-056

[B4] Karjalainen T Kellomäki S Greenhouse gas inventory for land use changes and forestry in Finland based on international guidelines Mitigation Adapt Strategies Global Climate 1996 n. 1 51 71

[B5] Weiss P Schieler K Schadauer K Radunsky k Englisch M Die Kohlenstoffbilanz des österreichischen Waldes und Betrachtungen zum Kyoto-Protokoll Series Die Kohlenstoffbilanz des österreichischen Waldes und Betrachtungen zum Kyoto-Protokoll 2000 Federal Environment Agency, Wien14560095

[B6] Kauppi PE Mielikäinen K Kuusela K Biomass and carbon budget of European forests, 1971-1990 Science 1992 256 70 74 10.1126/science.256.5053.70 17802594

[B7] Kauppi PE Kauppi E Tomppo E Ferm A C and N storage in living trees within Finland since 1950s Plant Soil 1995 168/169 633 638 10.1007/BF00029377

[B8] Löwe H Seufert G Raes F Comparison of methods used within member states for estimating CO_2 _emissions and sinks according to UNFCCC and EU monitoring mechanism: forest and other wooded land Biotechnol Agron Soc Environ 2000 4 315 319

[B9] UN-ECE/FAO Forest Resources of Europe, CIS, North America, Australia, Japan and New Zealand (industrialized temperate/boreal countries) UN-ECE/FAO Contribution to the Global Forest Resources Assessment 2000 2000 Main Report United Nations, New York, Geneva 2000 UN-ECE/FAO

[B10] FAO Global Forest Resource Assessment 2000 2001 FAO Forestry Paper 140. FAO, Rome

[B11] IPCC pIntergovernmental Panel on Climate Change Guidelines for National Greenhouse Gas Inventories 2006 http://www.ipcc.ch

[B12] Satoo T Madgwick HAI Forest biomass Forestry Sciences 1982 Martinus Nijhoff/Dr. W. Junk Publisher, The Hague 152

[B13] Bartelink HH Allometric relationships for biomass and leaf area of beech (*Fagus sylvatica *L.) Ann For Sci 1997 54 39 50 10.1051/forest:19970104

[B14] Ter-Mikaelian MT Korzukhin MD Biomass equations for sixty-five North American tree species For Ecol Manage 1997 97 1 24 10.1016/S0378-1127(97)00019-4

[B15] Lehtonen A Mäkipää R Heikkinen J Sievänen R Liski J Biomass expansion factors (BEFs) for Scots pine, Norway spruce and birch according to stand age for boreal forests Forest Ecology and Management 2004 188 1(3) 211 224

[B16] Levy PE Hale SE Nicoli BC Biomass expansion factors and root: shoot ratios for coniferous tree species in Great Britain Forestry 2010 77 5 421 430

[B17] Liski J Perruchoud D Karjalainen T Increasing carbon stocks in the forest soils of Western Europe For Ecol Manage 2002 169 163 179

[B18] Schroeder P Brown S Mo J Birdsey R Cieszewski C Biomass estimation for temperate broadleaf forests of the United States using inventory data For Sci 1997 43 424 434

[B19] Brown SL Schroeder PE Spatial patterns of aboveground production and mortality of woody biomass for Eastern US forests Ecol Applicat 1999 9 968 980

[B20] Brown S Measuring carbon in forests: current status and future challenges Environ Pollut 2002 116 363 372 10.1016/S0269-7491(01)00212-3 11822714

[B21] Fukuda M Iehara T Matsumoto M Carbon stock estimates for sugi and hinoki forests in Japan For Ecol Manage 2003 184 1 16 10.1016/S0378-1127(03)00146-4

[B22] Ishii H Mcdowell NG The role of epicormic branches in crown development of old Douglas-fir trees Forest Ecology and Management 2002 3 169 257 270

[B23] Binkley D Stape JL Ryan MG Barnard H Fownes J Age-related decline in forest ecosystem growth: an individual-tree, stand-structure hypothesis Ecosystems 2002 5 58 67 10.1007/s10021-001-0055-7

[B24] Vanninen P Ylitalo H Sievanen R Makela A Effects of age and site quality on the distribution of biomass in Scots pine (Pinus sylvestris L.) Trees - Structure and function 1996 10 4 231 238

[B25] Mäkelä AA Carbon balance model of growth and self-pruning in trees based on structural relationships Forest Science 1997 1 43 7 24

[B26] Li MH Krauchi N Dobbertin M Biomass distribution of different-aged needles in young and old Pinus cembra trees at highland and lowland sites Trees - Structure and function 2006 10 5 611 618

[B27] Nutto L Spathelf P Modelagem da desrama natural de *Araucaria angustifolia *(BERT.) O. KTZE Revista Floresta 2003 3 33 295 309

[B28] Ruel J Aires M Jensen's inequality predicts effects of environmental variation Tree 1999 14 9 10.1016/s0169-5347(99)01664-x 10441312

[B29] Rastetter EB Mckane RB Shaver GR Melillo JM Changes in C storage by terrestrial ecosystems: how C-N interactions restrict responses to CO2 and temperature Water, Air and Soil Pollution 1992 64 327 344

[B30] Magnani F Mencuccini M Grace J Age-related decline in stand productivity: the role of structural acclimation under hydraulic constraints Plant Cell Environ 2000 23 251 263 10.1046/j.1365-3040.2000.00537.x

[B31] Kira T Shidei R Primary production and turnover of organic matter in different forest ecosystems of the western Pacific Japanese Journal of Ecology 1967 17 1 70 87

[B32] Barnes BV Zak DR Denton SR Spurr SH Forest Ecology 1998 4 Wiley, New York 792

[B33] Ryan MG Binkley D Fownes JH Giardina CP Senock RS An experimental test of the causes of forest growth decline with stand age Ecological Monographs 2004 74 3 393 414 10.1890/03-4037

[B34] Ryan M Phillips N Bond BJ The hydraulic limitation hypothesis revisited Plant, Cell and Environment 2006 29 367 381 10.1111/j.1365-3040.2005.01478.x 17080592

[B35] Soares P Tomé M Hasenauer H, Makela A Analysis of the effectiveness of biomass expansion factors to estimate stand biomass Modeling Forest Production 2004 Proc. Conf. Vienna, 19-21 April (Department of Forest and Soil Sciences, BOKU University of Natural Resources and Applied Life Sciences, Vienna 368 374

[B36] FAO State of the world's forests 1997 1997 Food and Agricultural Organization of the United Nations 20021940926

[B37] Oliveira EB Um sistema computadorizado de prognose do crescimento e produção de Pinus taeda L., com critérios quantitativos para avaliação técnica e econômica de regimes de manejo 1995 (Tese - Doutorado em Ciências Florestais) - Setor de Ciências Agrárias, Universidade Federal do Paraná, Curitiba 134

[B38] Sette JRCR Nakajima NY Geromini MP Captura de carbono orgânico em povoamentos de *Pinus taeda *L. na região de Rio Negrinho, SC Revista Floresta 2006 36 1 Curitiba 33 44

